# Acute remote ischemic conditioning enhances (CD3+)- but not (FoxP3+)-T-cell invasion in the tumor center and increases IL 17 and TNF-alpha expression in a murine melanoma model

**DOI:** 10.3389/fimmu.2024.1501885

**Published:** 2024-11-22

**Authors:** Katarzyna Rachunek-Medved, Sabrina Krauß, Adrien Daigeler, Constantin Adams, Franziska Eckert, Katrin Ganser, Irene Gonzalez-Menendez, Leticia Quintanilla-Martinez, Jonas Kolbenschlag

**Affiliations:** ^1^ Department of Hand, Plastic, Reconstructive and Burn Surgery, BG Trauma Center, Eberhard Karls University of Tuebingen, Tuebingen, Germany; ^2^ Department of Paediatrics, University Hospital Tuebingen, Tuebingen, Germany; ^3^ Department of Radiation Oncology, University of Tuebingen, Tuebingen, Germany; ^4^ Institute of Pathology and Neuropathology, Comprehensive Cancer Center, University Hospital Tuebingen, Eberhard Karls University of Tuebingen, Tuebingen, Germany; ^5^ Cluster of Excellence iFIT (EXC2180) “Image-Guided and Functionally Instructed Tumor Therapies”, University of Tuebingen, Tuebingen, Germany

**Keywords:** remote ischemic conditioning (RIC), hypoxia, immunomodulation, tumor perfusion, melanoma (B16)

## Abstract

**Introduction:**

Hypoxia can drive tumor progression, suppress anti-tumor immunity, and reduce the effectiveness of radiotherapy and chemotherapy. This study aimed to assess the impact of remote ischemic conditioning (RIC) on tumor oxygenation (sO2) and the anti-tumor immune response.

**Material and methods:**

Fourteen B16-Ova tumor-bearing C57BL/6N mice received six 5-minute RIC cycles, while another fourteen underwent anesthesia only. Pimonidazole was administered 1.5 hours before sacrifice. Blood flow, sO2, and hemoglobin levels were measured in the non-ischemic hind limb and tumor. Tumor hypoxia was assessed using pimonidazole and CA IX immunohistochemistry, and T cell infiltration by CD3 and FoxP3 staining. Serum levels of 23 cytokines were analyzed using a multiplex immunoassay.

**Results:**

Isoflurane anesthesia caused a slight intraindividual increase in blood flow (p = 0.07) and sO_2_ (p = 0.06) of the hind limb and a sole increase in tumor sO_2_ (p = 0.035), whereas RIC improved sO_2_ of the tumor in relation to the hind limb (p=0.03). The median of the tumor oxygen saturation reached 51.4% in the control group and 62.7% in the RIC group (p = 0.09), exhibiting a slight tendency towards better oxygenation in the RIC group. Pimonidazole (p=0.24) and CA IX hypoxia score (p=0.48) did not reveal statistically significant differences between the two groups. In RIC-treated tumors, the number of CD3 (p=0.006), but not FoxP3- positive cells (p = 0.84), in the tumor core was significantly higher compared to the control group. In the RIC group, the mean fluorescence intensity (MFI) of IL-17 was significantly higher (p=0.035), and TNF-α was trend-wise higher (p=0.063) compared to the control group.

**Conclusion:**

Both isoflurane anesthesia and RIC have an impact on microcirculation. The application of RIC counteracted some of the effects of isoflurane, primarily in healthy tissue, and led to a significant improvement in relative tumor tissue oxygenation compared to the non-ischemic hind limb. RIC selectively enhanced immune infiltration within the tumor center, probably by previously activated tumor infiltrating T cells, while having no significant impact on T-regulatory cells. RIC appears to impact the cytokine profile, as indicated by elevated MFIs of TNF-α and IL-17.

## 1 Introduction

In their seminal paper, Hannahan and Weinberg defined sustained angiogenesis as one of the hallmarks of cancer (1–3). In solid tumors, however, angio- and vasculogenesis often lag behind proliferative tumor expansion, resulting in tumor hypoxia. Adaptation of tumor cells to a hypoxic tumor microenvironment (TME) is frequently associated with malignant tumor progression. Importantly, tumor hypoxia limits the access of chemotherapeutics to the tumor cells and lowers the efficacy of radiotherapy by a factor of 2-3 (4).

The oxygen deficiency negatively influences the immune response against the tumor via the transcriptional factor HIF-1α, which has been shown to activate the expression of PD-L1 on the surface of malignant cells in renal cell carcinoma (5, 6). HIF-1α regulates the expression of CA IX (carbonic anhydrase isoform 9) at the transcriptional level. CA IX contributes to the acidosis of the TME, which negatively influences the cytotoxic function of CD8-postive T cells and IFN production (7). Moreover, HIF-1α upregulates the expression of CD39 and CD73 on the tumor cells, contributing to the accumulation of adenosine in the TME (8). Ligation of adenosine receptors (A2aR and A2bR) on the surface of anti-tumor T cells subsequently triggers the upregulation of PD1 and CTLA4 on the T cell surface, resulting in the inhibition of IFN secretion and collectively culminating in the functional suppression of the anti-tumoral response (9–13). Additionally, cancer cells under hypoxia downregulate the expression of major histocompatibility complex (MHC) class I molecules (14). These molecules are essential for recognition by immune cells and immune-mediated lysis of tumor cells (15).

As early as 1989, it was recognized that the density of tumor-infiltrating lymphocytes (TILs) within primary cutaneous melanoma lesions holds prognostic significance for overall survival (16). In several subsequent studies, T-cell infiltration and function have been associated not only with improved overall survival rates in human melanoma but also with a better response to immunotherapies (17–22). In modern immunotherapy, developing new therapeutic strategies to reduce the hypoxic tumor area is therefore of great significance. These findings gave rise to the therapy approach of vessel normalization (23–25). This is based on the idea that the restoration of a more “physiological” tumor perfusion may hinder the negative direct influence of hypoxia on the tumor cell biology, simultaneously improving the anti-tumor immune response and increasing the treatment efficacy.

Remote Ischemic Conditioning (RIC) has been used in different clinical entities and *in vivo* experiments to achieve some of these goals (26–34). The realization that brief, non-detrimental periods of ischemia prior to an ischemic insult confer a protective effect on tissue traces its origins to the work by Murry et al. in 1986 (27). In an animal model, they demonstrated that the extent of tissue damage caused by a coronary infarction can be substantially mitigated when the infarction is preceded by several short cycles of ischemia and reperfusion (I/R) within the same vascular region. A few years later, the concept of local ischemic conditioning was expanded upon by Przyklenk et al. (35, 36) They discovered that brief ischemic episodes in an anatomically separate coronary vascular bed of the heart also conferred remote protection upon non-directly preconditioned myocardium against subsequent sustained coronary artery occlusion. Building upon these insights, it was demonstrated that this effect is not confined solely to the heart but is systemically transferable to other organs.

Brzozowski et al. investigated the impact of cardiac, hepatic, and gastric preconditioning using brief ischemia (occlusion of coronary arteries as well as hepatic and celiac arteries twice for 5 minutes each) on gastric blood flow and damage following a 3-hour period of ischemia/reperfusion (37). A 3-hour ischemia followed by reperfusion of the stomach resulted in numerous gastric lesions, decreased gastric blood flow, reduced mucosal prostaglandin E2 (PGE2) production, and elevated expression and release of IL-1 and TNF-α. These authors demonstrated that remote ischemic preconditioning could mitigate the deleterious consequences of ischemia. Since the protective effects of brief gastric, cardiac, and hepatic preconditioning were nullified by selective cyclooxygenase-1 and cyclooxygenase-2 inhibitors, as well as by capsaicin denervation, these authors hypothesized that the mechanism of RIC preconditioning was based on the action of prostaglandins produced by cyclooxygenase-1 and cyclooxygenase-2, along with the activation of sensory nerves releasing calcitonin gene-related peptide (CGRP) (37).

Koike et al. investigated the influence of remote ischemic conditioning on the development and progression of experimentally induced necrotizing enterocolitis (NEC) in a murine model (26). Applying RIC to the hind limb of newborn mice with induced NEC improved blood circulation to the immature intestine due to vessel dilation mediated by nitric oxide and hydrogen sulfide and positively influenced survival.

While being investigated in a variety of conditions, the impact of RIC on tumor perfusion and antitumor immune response in malignancies has, at least to our knowledge, not yet been studied. Therefore, this work aims to investigate the effects of RIC on tumor perfusion, immune cell infiltration and cytokine profile in an orthotopic, syngeneic melanoma mouse model with B16-OVA melanoma cells and C57BL/6NCrl mice.

## 2 Material and method

The animal experiments were authorized by the regional board of Baden-Wuerttemberg (Project number BG 01/19G) and conducted in accordance with the national guidelines and regulations.

### 2.1 Animals

To analyze hypoxia and the immune response due to RIC, an orthotopic, syngeneic melanoma murine model with B16-OVA cells and 6–8-week-old C57BL/6NCrl mice was chosen.

Due to the biometric experimental design, 14 animals per group were required (38). Considering a tumor engraftment rate of approximately 90%, two additional mice per group were allocated as reserves. The study will include 6-8-week-old C57BL/6NCrl (Charles River) mice (equal numbers of males and females) with a weight of approximately 25 g. All mice included in the experiments were maintained in “Type 2 long” individually ventilated cages (IVC) under specified pathogen-free conditions, segregated by gender. The animals were provided with ample cellulose for nest-building, wood shavings, a shelter or wooden tubes, and ad libitum access to food and water. The acclimatization phase lasted a minimum of 9 to 14 days.

### 2.2 B16-OVA cell-line

The B16-OVA cells were obtained from IDEXX GmbH laboratory (Ludwigsburg, Germany) and tested negative for the following pathogens using PCR (IMPACT II PCR Profile) prior to the experimental series: Mycoplasma species, Mouse hepatitis virus (MHV), Minute virus of mice (MVM), Mouse parvovirus (MPV1-5), Theiler’s murine encephalomyelitis virus (TMEV), Sendai virus, Pneumonia virus of mice (PVM), Murine norovirus (MNV), Reovirus 3 (REO3), Mouse rotavirus (EDIM), Ectromelia virus, Lymphocytic choriomeningitis virus (LCMV), Polyomavirus, Lactate dehydrogenase-elevating virus (LDEV), Mouse adenovirus (MAV1), Mouse adenovirus (MAV2), and Mouse cytomegalovirus (MCMV). The B16-OVA cell line was cultured in Roswell Park Memorial Institute (RPMI) 1640 medium (Gibco), supplemented with 10% fetal bovine serum (FBS, Gibco), 1 mM sodium pyruvate, and 2 mM L-glutamine. The cells were maintained in a humidified incubator at approximately 37°C and 5% CO2 concentration. Cell culture was passaged every 4-5 days.

### 2.3 Implantation of B16-OVA cells

Syngeneic B16-OVA cells [1,5 × 10^5^ cells in 0.5 ml phosphate-buffered saline (PBS), cell viability >90%] were implanted subcutaneously in the right flank of the mice. Once the tumor had reached the size of 0.7 to 1 cm in diameter, the RIC/sham procedure was performed. Inclusion to the RIC or control group was based on a stratified randomization by sex.

### 2.4 Remote ischemic conditioning

The mice were anesthetized with isoflurane and placed on a heat pad. The core temperature of the mice was measured every 5 minutes. Before applying RIC, a 2-minute baseline measurement of the perfusion parameters was conducted. In the RIC group, six 5-minute RIC cycles were conducted with a reperfusion phase of 5 minutes following each RIC cycle. To achieve reproducible results and to minimize the pressure applied on the hind limb of the mouse, the low-pressure tourniquet designed for the C57BL/6 mouse strain by Drysch et al. was applied as follows: “A cord was attached to an aluminum plate on the one side and to a newton meter on the other side while the newton meter was attached to a hook. The hook could be moved freely on a splint in order to precisely reproduce the necessary amount of force. To induce ischemia, the cord was wrapped around the hind limb, and (…) a force of 0.6 N was used to cause occlusion of the vessels.” (39) The ischemia was confirmed by inspection (pallor of the hind limb and hyperemia after removal of the tourniquet) and with a pulse oximeter (hemoglobin O_2_ saturation, sO_2_<80%) at the end of the first RIC phase. The animals in the control group were placed under isoflurane anesthesia for 57 minutes under the same conditions but without applying RIC.

### 2.5 Microcirculation

We used a combined laser Doppler and white light spectroscopy device (O2C, LEA Medizintechnik GmbH) to non-invasively analyze the microcirculation, including blood flow, oxygen saturation of hemoglobin SO_2_[%] and relative hemoglobin amount (rHb) in the examined tissue. Probes were applied on the tumor and, for control, on the right non-ischemic hind leg of the mouse. The measurements were conducted in real-time during the experiment.

### 2.6 Tumor tissue and blood samples

To determine the hypoxic tumor areas, the animals of both groups were given pimonidazole (100 mg/kg) intraperitoneally after the last reperfusion phase in the RIC group or after 57 min of anesthesia in the sham group. Ninety minutes after the pimonidazole injection, animals were sacrificed by cervical dislocation, and after blood was collected retrobulbarly. Afterward, tumors were excised and fixed in 4% formalin.

The collected blood was left in Eppendorf tubes at room temperature to allow coagulation (approximately 20 minutes). Subsequently, a centrifugation step was performed at 10,000 x g for 15 minutes at 4°C. The serum was then transferred to a fresh tube and stored in a freezer at -80°C.

### 2.7 Histological analysis

Formalin-fixed tumors were embedded in paraffin. For histology, 3-5 µm-thick sections were cut and stained with hematoxylin and eosin (H&E). Immunohistochemistry was performed on an automated immunostainer (Ventana Medical Systems, Inc.) according to the company’s protocols for open procedures. The slides were stained with the antibodies CD3 (Clone SP7, DCS Innovative Diagnostik-Systeme GmbH u. Co. KG, Hamburg, Germany), MAb1 (Hypoxyprobe, Burlington, Massachusetts, USA), CA IX (Abcam plc, 330 Cambridge Science Park, Cambridge, UK) and FoxP3 (Abcam plc, 330 Cambridge Science Park, Cambridge, UK). Appropriate positive and negative controls were used to confirm the adequacy of the staining. All samples were scanned with the Ventana DP200 (Roche, Basel, Switzerland) and processed with the Image Viewer MFC Application. Final image preparation was performed with Adobe Photoshop CS6.

#### 2.7.1 Necrosis

The area of the tumor and necrosis was measured on the scanned H&E slides. Subsequently, the percentage of necrosis was calculated relative to the total tumor area.

#### 2.7.2 Hypoxia

CA IX is an endogenous hypoxia marker in both human and murine tissue. *In vitro*, CA IX requires six hours to reach maximum expression under anoxic conditions. The half-life of CA IX ranges from 38 to over 96 hours, resulting in slow responsiveness to reoxygenation and the ability to indicate chronic tumor hypoxia (40). In contrast, Pimonidazole staining rapidly responds to changes in tumor hypoxia and hypoxia-modifying interventions due to its direct redox reaction in hypoxic tissue (40, 41).

Given the diffuse nature of hypoxia itself, the boundaries of hypoxic areas cannot be precisely quantified. Therefore, we have established a grading system for a semi-quantitative analysis of hypoxia:

+: A few small hypoxic areas
++: moderate hypoxic areas
+++: large hypoxic areas

#### 2.7.3 Infiltration by T cells

Finally, the inflammatory infiltrate in both groups was examined using immunohistochemistry with anti-CD3 antibodies. The analysis of CD3-positive T-cell infiltration was separately conducted for the tumor periphery and the tumor center. Quantitative analysis of the center’s infiltration was performed by counting CD3-positive cells at 400x magnification in a total of 10 high-power fields, from which an average value was derived. Compared to the tumor center, the tumor periphery exhibits significant heterogeneity. Thus, to assess T-cell infiltration, a semi-quantitative analysis was established. The entire tumor periphery was observed at 400x magnification and classified using the scale described below:

-: no detectable T cells
+: A few T cells
++: moderate T-cell infiltration
+++: strong T-cell infiltration

In cases of borderline findings, two values can be indicated (e.g., +/++, ++/+++, -/+).

#### 2.7.4 Infiltration by regulatory T cells

Treg cells are identified through immunohistochemistry using anti-FoxP3 antibodies. In the tumor center, quantification of FoxP3-positive cells was performed in 10 randomly selected high-power fields (at 200x magnification), from which an average value was calculated. Additionally, the amount of FoxP3-positive cells at the tumor edge was evaluated as follows:

-: negative,
+: low, and
++: mild.

### 2.8 Multiplex immunoassay

After thawing the serum samples, which were stored at -80°C, centrifugation at 10,000 x g for 10 minutes was performed. The Bio-Plex^®^ Multiplex Immunoassay of the serum samples was conducted with The Bio-Plex^®^ 200 system and a dedicated Bio-Plex^®^ Pro Mouse Cytokine 23-Plex Assay Kit (Bio-rad). The preparation and dilution of serum samples, and the assay were performed in accordance with the producer guidelines in duplicates.

### 2.9 Statistical method

Statistical analyses were conducted using SAS software. The Shapiro-Wilk test was employed to assess the distribution of metric variables among independent groups. In instances where the normality assumption was rejected, the Mann-Whitney U test (MWU) was utilized for comparisons between two independent samples, while the Wilcoxon signed-rank test was applied for dependent samples. To evaluate the frequency distributions of a categorical variable among independent groups, Fisher’s exact test was implemented. The correlation between the two datasets was assessed using Pearson’s coefficient for metric variables and Spearman’s coefficient for ordinal and metric variables. All tests were conducted bilaterally. Given the exploratory nature of the analysis, p-values are interpreted descriptively.

## 3 Results

A total of 28 (out of 32) animals were included in the experiment; 14 were in the control group and 14 in the RIC group. The sex distribution in both groups was the same, with 7 male and 7 female C57BL/6N mice. Three animals reached termination criteria prematurely and had to be excluded. Engraftment of tumors was observed in 31 out of 32 B16-OVA-transplanted mice.

The experiment was initiated in the control and RIC group at a median tumor diameter of 8.60 mm and 8.88 mm (p=0.85), at a median of 19 and 18 days, respectively, (p=1) after the implantation of the tumor cells (**Table 1**). Neither age (median of 11 and 12 weeks respectively in control and RIC group, p=0.66), body weight (median of 22.8 g and 23.8 g, respectively p=0.65) nor core temperature during the experiments (37°C in both groups, p=0.94) differed between the two groups (**Table 1**).

**Table 1:** Presentation of the basic data of the studied population and individual results of the histopathological analysis in the control (Co.) and RIC groups.

Group	Age (weeks)	Weight (g)	Tumor diameter (mm)	Necrosis (%)	Average number of CD3^+^ cells/HPF, tumor center	CD3^+^ cells in the tumor periphery	Average number of Tregs/HPF, tumor center	Tregs in the tumor periphery	MAb1	CA IX
Co.1	9.5	25.3	10.5	20.52	3.1	+/++	1.8	+	++	+++
Co. 2	9.5	25.1	11	11.03	0.8	+	0.96	+/++	+	+++
Co. 3	9.5	19.4	8.35	6.49	1.7	+	1.0	+	+	+++
Co. 9	11.5	22	8.5	43.09	1.2	+	0.75	+	+++	+++
Co. 13	9	22.5	8.5	9.24	1.8	+/++	2.45	+	++	+++
Co. 16	14	23.3	8.5	47.81	1.5	++/+++	0.67	+	++	+++
Co. 18	9.5	23	9.5	54.41	1.8	+	0.2	-/+	++	+++
Co.19	14.5	26.6	10.0	31.36	3.7	++/+++	0.22	+	+++	+++
Co. 20	10	20	7.25	30.92	1.9	++/+++	2.75	++	++	+++
Co. 22	10.5	24.5	8.7	36.73	1.8	++	1.18	+/++	+++	+++
Co. 24	11	20.1	7.5	8.42	1.5	+	0.21	-/+	++	+++
Co. 26	11.5	20.5	8.5	11.1	1.5	++	0.35	+	+	+++
Co. 27	12	22.2	9.25	9.66	1.1	+/++	0.0	-/+	++	+++
Co. 29	13.7	29	9.0	32.23	1.0	+/++	0.12	-/+	+	+++
RIC 4	9.5	19.8	9.5	14.65	2.9	+	0.0	-/+	++	+++
RIC 6	10	22	8,0	24.69	1.4	++	0.27	+	+++	+++
RIC 7	12	26.8	10	36.62	2.2	++	0.74	+	+++	+++
RIC 8	13	28	9.0	32.7	5.5	++/+++	1.25	+/++	+++	+++
RIC 10	12	22.8	9.5	23.59	1.8	-/+	0.4	-/+	+++	+++
RIC 11	12	24.8	15.0		3.7	+/++	1.21	++	+++	++
RIC 12	12	24.8	8.75		4.7	++/+++	5.33	++	++	++
RIC 14	9	19	7.75	39.84	3.1	+	0.25	-/+	+++	+++
RIC 15	9	21.5	8.5	27.1	2.8	++/+++	0.44	-/+	+++	+++
RIC 17	14	27.1	8.0	6.45	4.5	+++	0.0	+/++	+	+++
RIC 21	10	21.6	9.0	24.86	4.1	++/+++	1.1	+/++	++	+++
RIC 23	11	20.4	7.5	6.86	2.0	+	0.3	+	+	+++
RIC 25	11.5	26.7	9.25	31.74	2.0	+/++	0.41	+	+	+++
RIC 28	13.5	28	7.2	60.13	1.1	+/++	0.59	+	+	+++

Two RIC samples were not considered for necrosis area measurement because the samples were partially fragmented.

The symbols “+”, “++”, “+++”, and “-” represent a semi-quantitative grading system for hypoxia and T-cell infiltration. For hypoxia, + indicates a few small areas, ++ moderate, and +++ large hypoxic areas. For T-cell infiltration, - means no detectable T cells, + a few, ++ moderate, and +++ strong infiltration. FoxP3-positive cells at the tumor edge are classified as - (negative), + (low), and ++ (mild). In cases of borderline findings, two values can be indicated (e.g., +/++, ++/+++, -/+).

### 3.1 Microcirculation measurements

#### 3.1.1 Comparison of perfusion parameters between the RIC and control groups during baseline measurement

**Figure 1:**
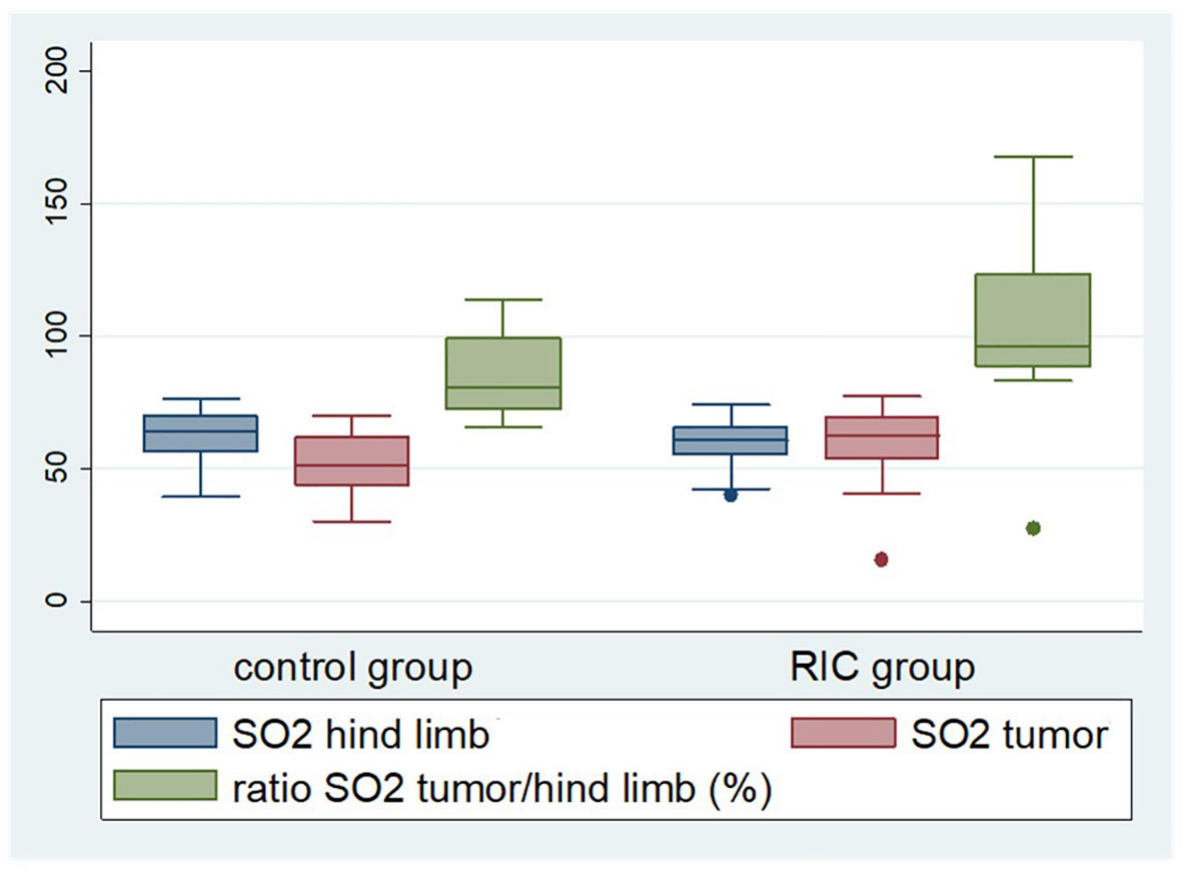
Box plot displaying selected perfusion parameters, namely tissue oxygenation (SO2) of the tumor and non-ischemic hind limb, as well as tumor tissue oxygenation in relation to the oxygen saturation of the non-ischemic hind limb during the last reperfusion phase. The latter was significantly higher in the RIC group (median 96%) compared to the control group (median 82%) (Mann-Whitney U test, p = 0.03). The median of the tumor oxygen saturation reached 51.4% in the control group and 62.7% in the RIC group (Mann-Whitney U test, p = 0.09), exhibiting a slight tendency towards better oxygenation in the RIC group.

The median tissue blood flow (measured in arbitrary units [AU]) during the baseline phase at the hind paw was 134.7 AU in the control group and 129.4 AU in the RIC group, showing no statistically significant differences (MWU, p = 0.77). The median blood flow in tumor tissue reached 204.0 AU in the control group and 157.7 AU in the RIC group. These differences were also not statistically significant (MWU, p = 0.57) (**Table 2**, **Figure 1**).

**Table 2:** Overview and comparison of perfusion parameters in the baseline phase in the control and RIC groups, including mean, standard deviation (STD), minimum (Min), median, and maximum (Max) values, as well as p-values from the statistical analysis (Mann-Whitney U test).

	Group	Number	Mean	STD	Min	Median	Max	p-value
Flow hind limb (Baseline)	control	14	141.6	47.7	83.0	137.4	240.0	0.77
	RIC	14	145.7	40.8	97.9	129.4	222.5	
Flow tumor (Baseline)	control	14	178.9	70.5	35.1	204.0	258.5	0.57
	RIC	14	171.6	88.3	45.7	157.7	328.8	
SO_2_ hind limb (Baseline)	control	14	55.6	11.7	34.2	52.3	75.7	0.12
	RIC	14	61.3	9.3	48.7	59.4	81.3	
SO_2_ Tumor (Baseline)	control	14	47.1	15.7	13.3	49.4	72.3	0.15
	RIC	14	55.8	14.2	22.3	56.5	73.1	
rHb hind limb (Baseline)	control	14	78.4	9.2	64.4	79.6	93.3	0.60
	RIC	14	80.2	7.6	65.3	80.6	90.4	
rHb Tumor (Baseline)	control	14	52.1	21.4	23.4	49.2	84.4	0.51
	RIC	14	58.4	19.6	24.5	62.1	83.1	

Oxygen saturation in the non-ischemic paw did not show significant differences between the groups at the onset of the experiment, with a median of 52.3% in the control group and 59.4% in the RIC group (MWU, p = 0.12). Similarly, tumor oxygen saturation did not differ significantly between the two groups, with medians of 49.4% in the control group and 56.5% in the RIC group (Mann-Whitney U test, p = 0.15) (**Table 2**, **Figure 1**).

The median rHb of the non-ischemic paw, measured in “relative absorbance units” (RAU), reached 79.6 RAU in the control group and 80.6 RAU in the RIC group, respectively (MWU, p = 0.60). The rHb of the tumor during the baseline phase reached a median of 49.2 RAU in the control group and 62.1 RAU in the RIC group, showing no statistically significant differences (MWU, p = 0.51) (**Table 2**, **Figure 1**).

#### 3.1.2 Impact of isoflurane anesthesia (control group)

Isoflurane anesthesia might potentially impact perfusion parameters. For this reason, these parameters were initially analyzed within the control group. The values between the endpoint and baseline phases were compared for the non-ischemic paw and tumor tissue.

In healthy tissue (at the non-ischemic paw), a slight increase in flow (p = 0.07) and oxygen saturation (p = 0.06) was observed (**Table 3**). This could have resulted from the mild vasodilative effect of isoflurane. In contrast, within the tumor tissue, only oxygen saturation shows an increase (p = 0.035), while other parameters remained unchanged (**Table 4**). As flow and rHb remained stable, it can be hypothesized that isoflurane anesthesia might have led to reduced oxygen extraction in the tumor tissue, thus resulting in a decrease in oxygen consumption. Isoflurane anesthesia appears to exert an influence on perfusion parameters even within the control group.

**Table 3:** Overview of perfusion parameters measured at the non-ischemic paw in the control group, and comparison between baseline and endpoint phases with corresponding minimum (Min) and maximum (Max) values, means, medians, standard deviation (STD), 25th and 75th percentiles, and p-values from the Wilcoxon test.

Flow hind limb			SO_2_ hind limb		rHb hind limb	
	Baseline	Last reperfusion	Baseline	Last reperfusion	Baseline	Last reperfusion
Min	82.97	43.55	34.16	39.50	64.35	48.19
Max	239.95	284.53	75.74	76.42	93.32	89.25
Mean	141.63	157.15	55.57	61.59	78.42	77.33
STD	47.65	61.39	11.69	10.41	9.18	11.20
Median	137.36	143.86	52.30	64.08	79.62	81.16
25^th^Percentile	103.40	122.47	48.07	56.03	69.44	70.86
75^th^ Percentile	172.74	186.99	67.40	70.13	85.41	85.16
p-value (Wilcoxon Test)	0.073		0.06		0.64	

**Table 4:** Overview of perfusion parameters measured in the tumor area within the control group, and comparison between baseline and endpoint phases with corresponding minimum (Min) and maximum (Max) values, means, medians, standard deviation (STD), 25th and 75th percentiles, and p-values from the Wilcoxon test.

	Flow Tumor		SO_2_ Tumor		rHb Tumor	
	Baseline	Last reperfusion	Baseline	Last reperfusion	Baseline	Last reperfusion
Min	35.1	54.03	13.32	29.97	23.35	34.21
Max	258.45	314.52	72.27	70.03	84.42	79.16
Mean	178.94	195.20	47.12	52.39	52.05	52.32
STD	70.47	67.51	15.71	12.42	21.41	16.97
Median	203.98	199.97	49.38	51.43	49.15	45.47
25^th^ Percentile	108.72	161.76	37.33	42.63	31.83	38.25
75^th^ Percentile	242.51	233.78	56.12	62.46	72.94	70.51
p-value (Wilcoxon test)	0.12		0.035*		0.27	

A statistically significant difference in tumor oxygen saturation is indicated with an asterisk (*).

#### 3.1.3 Comparison of perfusion parameters between the RIC and control groups during the last reperfusion phase

During the final reperfusion phase, the median tissue blood flow of the non-ischemic hind limb was 143.9 AU in the control group and 132.6 AU in the RIC group, showing no statistically significant differences (MWU, p = 0.48) (**Table 5**). In the tumor tissue, the median blood flow reached 200.0 AU in the control group and 179.6 AU in the RIC group, respectively (MWU, p = 0.66) (**Table 5**).

**Table 5:** Overview and comparison of perfusion parameters from the endpoint phase in the control and RIC groups, including mean, standard deviation (STD), minimum (Min), maximum (Max), median values, and p-values from the statistical analysis (Mann-Whitney U test).

	Group	Number	Mean	STD	Min	Median	Max	p-value
Flow hind limb	control	14	157.2	61.4	43.6	143.9	284.5	0.48
	RIC	14	138.6	38.8	84.6	132.6	201.0	
Flow tumor	control	14	195.2	67.5	54.0	200.0	314.5	0.66
	RIC	14	183.0	83.8	60.5	179.6	363.1	
SO_2_ hind limb	control	14	61.6	10.4	39.5	64.1	76.4	0.57
	RIC	14	59.1	10.6	40.2	60.7	74.1	
SO_2_ tumor	control	14	52.4	12.4	30.0	51.4	70.0	0.09
	RIC	14	59.7	16.0	15.7	62.7	77.3	
rHb hind limb	control	14	77.3	11.2	48.2	81.2	89.3	0.42
	RIC	14	70.7	17.8	29.2	74.6	89.8	
rHb tumor	control	14	52.3	17.0	34.2	45.5	79.2	0.42
	RIC	14	58.9	19.5	26.2	64.0	87.8	

The oxygen saturation of the non-ischemic limb did not exhibit significant differences during the last reperfusion phase of the experiment, with a median of 64.1% in the control group and 60.7% in the RIC group (MWU, p = 0.57). The median of the tumor oxygen saturation reached 51.4% in the control group and 62.7% in the RIC group (MWU, p = 0.09), exhibiting a slight tendency towards better oxygenation in the RIC group in the direct comparison (**Table 5**).

The differences in the median rHb of the non-ischemic paw between the control (81.2 RAU) and RIC group (74.6 RAU) were not statistically significant (MWU, p = 0.42). The rHb of the tumor reached a median of 45.5 RAU in the control group and 64 RAU in the RIC group during the last reperfusion phase, with no statistically significant differences (MWU, p = 0.42) (**Table 5**).

To further determine the effect of RIC on microcirculation of the ectopic tumors, the differences in the following parameters between the baseline and last reperfusion phase in the RIC group and the corresponding time in controls were compared. We did not detect statistically significant differences in the increase - delta (Δ) - of the perfusion parameters between the RIC and control groups (**Table 6**).

**Table 6:** Comparison of the chosen microcirculatory parameters and ratios between RIC and control groups with corresponding averages, standard deviations (SD), minimum (Min) and maximum (Max) values, median, and p-values of the Mann-Whitney-U test for the compared measurements.

Variable	Group	Number	Average	STD	Min	Median	Max	p-value
*Flow ratio tumor/ hind limb (baseline phase)*	Control	14	1.37	0.69	0.42	1.43	2,49	0.54
	RIC	14	1.20	0.63	0.47	1.02	2,56	
*sO_2_ ratio tumor/hind limb (baseline phase)*	Control	14	0.85	0.29	0.39	0.81	1,57	0.35
	RIC	14	0.92	0.27	0.42	0.89	1,50	
*rHb ratio tumor/hind limb (baseline phase)*	Control	14	0.67	0.30	0.30	0.68	1,19	0.45
	RIC	14	0.72	0.23	0.31	0.75	0,98	
*Flow ratio tumor/ hind limb (last reperfusion phase)*	Control	14	1.36	0.50	0.41	1.42	2,07	0.7
	RIC	14	1.40	0.77	0.59	1.15	2,83	
sO_2_ ratio tumor/hind limb (last reperfusion phase)	Control	14	0.85	0.16	0.66	0.81	1.14	0.03*
	RIC	14	1.03	0.32	0.27	0.96	1.68	
*rHb ratio tumor/hind limb (last reperfusion phase)*	Control	14	0.70	0.26	0.43	0.56	1.12	0.16
	RIC	14	0.86	0.33	0.46	0.79	1.67	
*Δ flow hind limb*	Control	14	15.53	29.84	-39.42	22.92	46.72	0.11
	RIC	14	-7.17	44.96	-94.03	7.30	63.19	
*Δ flow tumor*	Control	14	16.26	35.98	-47.65	17.91	85.48	0.7
	RIC	14	11.36	33.39	-44.33	12.95	78.76	
*Δ sO_2_ hind limb*	Control	14	6.02	9.92	-9.98	4.55	22.42	0.15
	RIC	14	-2.20	13.14	-36.00	2.70	10.54	
*Δ sO_2_ tumor*	Control	14	5.27	8.95	-6.18	1.06	23.97	0.87
	RIC	14	3.91	8.37	-6.56	3.14	22.81	
*Δ rHb hind limb*	Control	14	-1.09	6.10	-17.68	0.83	6.07	0.32
	RIC	14	-9.44	16.51	-45.65	-1.38	5.60	
*Δ rHb tumor*	Control	14	0.27	12.99	-37.00	3.00	16.68	0.95
	RIC	14	0.58	10.51	-17.86	1.87	18.20	

A statistically significant result is indicated with an asterisk (*).

#### 3.1.4 Tumor microcirculation in relation to normal tissue

In addition to the direct comparison of pre- and post-interventional perfusion parameters, it is important to provide a real-time assessment of tumor microcirculation in relation to normal tissue. Tissue microcirculation is influenced by multiple factors every second, depending on the current function of the circulatory system and tissue metabolism. To address this particular dynamic and the interplay of perfusion, the ratios described below were calculated in the baseline and endpoint phases and compared between the control and RIC groups.

In the baseline phase, there were no relevant differences between the control and RIC groups in the flow, oxygenation and rHb ratios (**Table 6**). The Flow tumor/non-ischemic paw ratio and the rHb tumor/non-ischemic paw ratio did not differ in the last reperfusion phase. However, the tumor tissue oxygenation in relation to the oxygen saturation of the non-ischemic hind limb during the last reperfusion phase was significantly higher in the RIC group compared to the control group (**Figure 1**). The median tumor oxygenation was 81% of the oxygen saturation of the non-ischemic paw in the control group, compared to 96% in the RIC group (MWU, p = 0.03) (**Table 6**). This suggests that the RIC intervention might influence the relative oxygen saturation in the venous-capillary vascular bed of the tumor in relation to normal tissue. This seems to occur without relevant changes in relative blood flow and relative hemoglobin content.

### 3.2 Histological analysis

#### 3.2.1 Necrosis

The measured necrosis area reached a median of 25.7% in the control group and 26% in the RIC group and did not show a statistically relevant difference (MWU, p=0.82) (**Table 1**).

#### 3.2.2 Hypoxia

**Figure 2:**
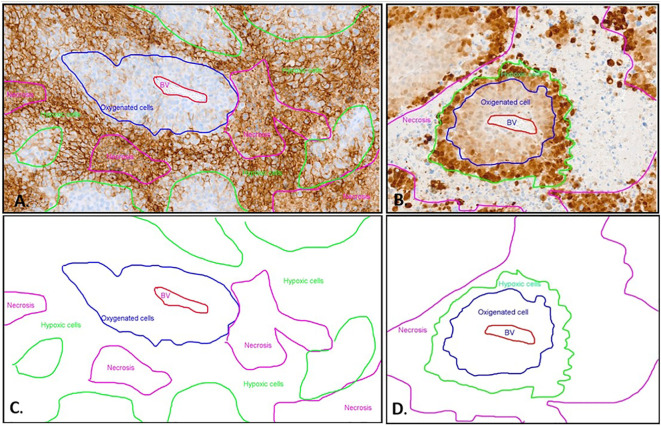
Immunohistochemistry with CA IX **(A)** and Pimonidazole **(B)** staining and schematic representation **(C, D)** of different oxygenated cells in relation to the blood vessel. **(A)** The CA IX-positive cells exhibit distinct membrane staining. The non-hypoxic tumor cells (blue line) are identified around a central blood vessel (BV, red marking). The majority of positive hypoxic cells (green line) are detected as palisade formations around the necrotic areas (pink line). However, in the CA IX staining, the necrosis also displays non-specific diffuse staining that is not associated with the membrane. This non-membrane-specific staining allows differentiation between the necrotic areas and the hypoxic cells. **(B)** The MAb1 immunohistochemistry displays a distinct hypoxic pattern. The non-hypoxic tumor cells (blue line) are identified around a central blood vessel (BV, red marking). Further away from the center of the blood vessel, the hypoxic tumor cells (green line) exhibit strong nuclear and cytoplasmic staining, forming a palisade between the oxygen-rich tumor cells and the necrosis (pink line). The necrosis shows no Pimonidazole (MAb1) staining. **(C)** Schematic depiction of distinct oxygenated cell distributions relative to the blood vessel in CAIX immunohistochemistry, using the same sample as presented in image A. **(D)** Schematic distribution of oxygenated cells in relation to the blood vessel in pimonidazole immunohistochemistry, employing the identical sample displayed in image B.

**Figure 3:**
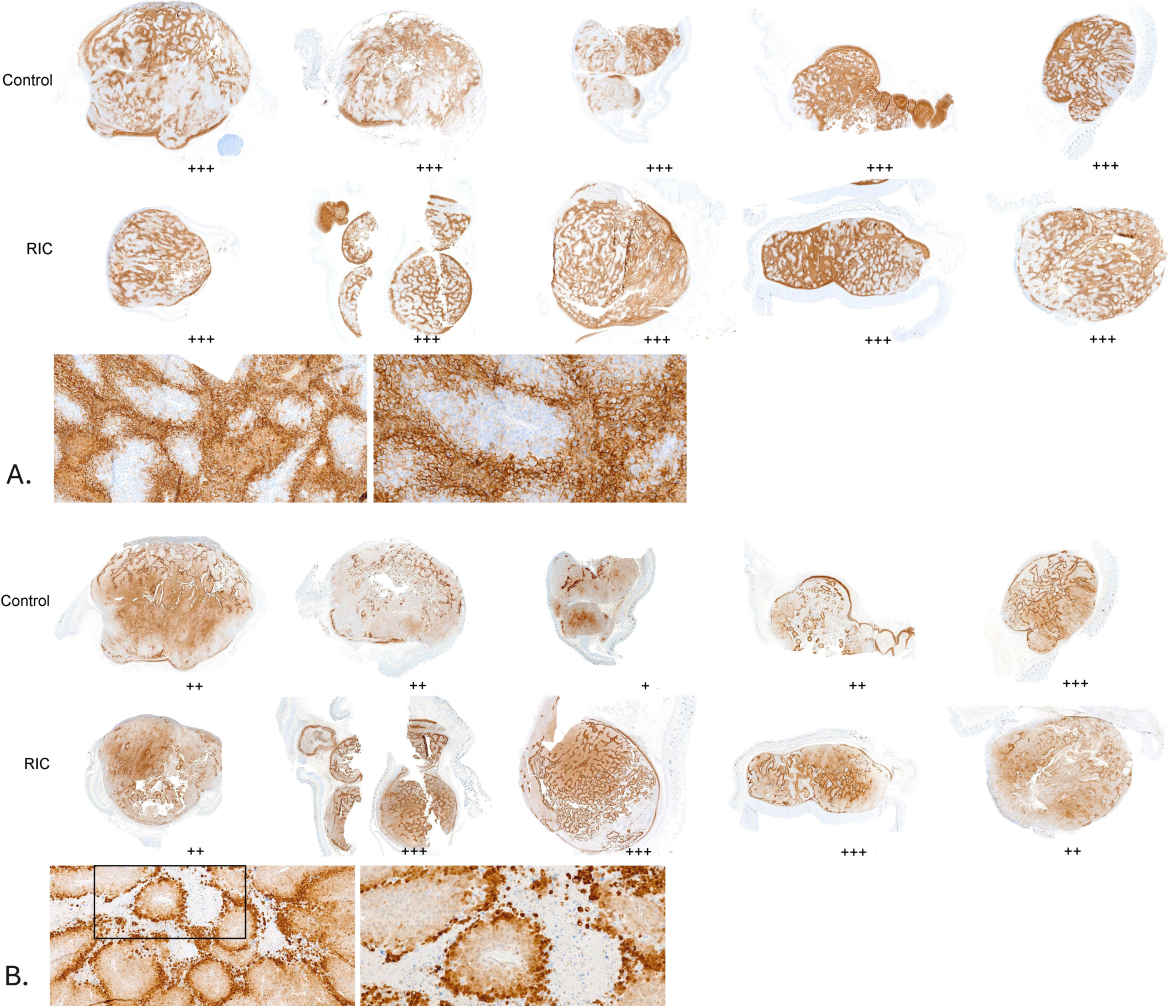
Hypoxia assessment in a series of tumor samples from the control and RIC groups. Hypoxic areas were evaluated using a semi-quantitative scoring system (+: few small hypoxic areas, ++: moderate hypoxic areas, +++: large hypoxic areas). No statistically significant differences were found between the RIC and control groups, either for pimonidazole staining (Fisher’s exact test, p = 0.24) or for CA IX staining (Fisher’s exact test, p = 0.48). **(A)** CA IX staining in tumor samples, with corresponding histological assessment of hypoxia using a semi-quantitative score. Sections of the tumor samples are shown at higher magnifications (10x and 20x) for better visualization. **(B)** Pimonidazole staining in tumor samples, with corresponding histological assessment of hypoxia using a semi-quantitative score. Sections of the tumor samples are shown at higher magnifications (10x and 20x) for better visualization.

The hypoxia was revealed by the MAb1 antibody (pimonidazole technique, acute hypoxia) and the CA IX antibody for chronic endogenous hypoxia (**Figure 2**). According to MAb1 staining, few hypoxic areas (+) were found in 4 RIC and 4 control group probes, whereas moderate acute hypoxia (++) was present in 7 RIC and 3 control probes (**Figure 3**). Large hypoxic areas (+++) were observed in 7 RIC and 3 control samples. These differences were not statistically significant (exact Fisher’s test, p=0.24). Indeed, no differences between groups were detected regarding the CA IX hypoxia score, with large hypoxic areas in almost all samples analyzed (control: +++ n=14; RIC: ++ n=2, +++ n=12) (exact Fisher’s test, p=0.48) (**Figure 3**). The intraindividual discrepancies between CA IX and MAb1 scores did not differ significantly between RIC and control groups (exact Fisher’s test p=0.13).

#### 3.2.3 Immune infiltration

##### 3.2.3.1 CD3- positive cells

**Figure 4:**
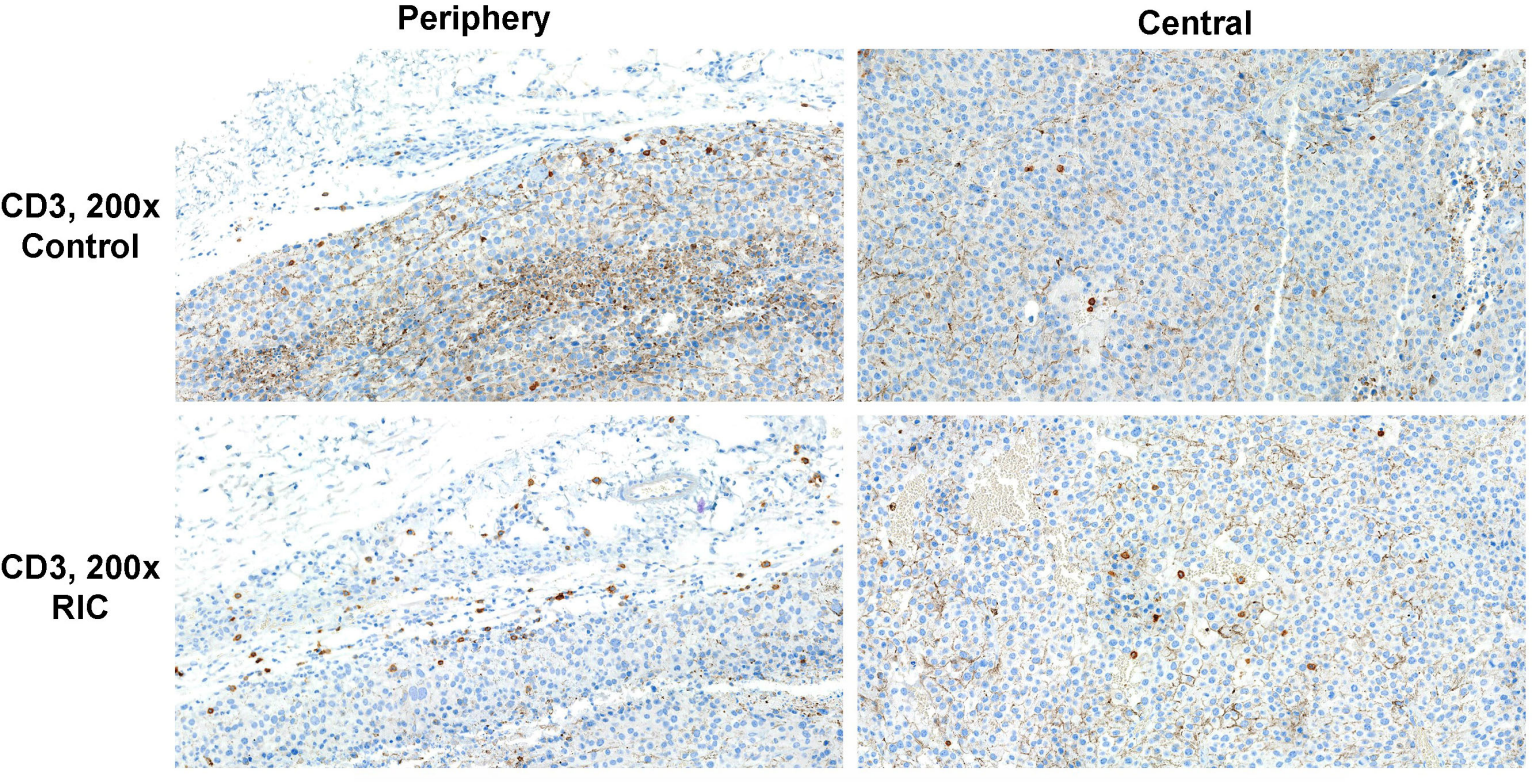
CD3 immunohistochemistry of tumor specimens presented at 200x magnification. CD3-positive cells are identifiable by brown staining and small cell nuclei. Moderate T-cell infiltration was observed in the tumor periphery of both the RIC and control group samples. Sparse immune infiltration was identified in the center of a tumor sample from the control group, in contrast to moderate immune infiltration with T cells in the center of a RIC tumor specimen. In RIC tumors, CD3-positive cell infiltration in the tumor center (median 2.8 T cells/400x HPF) was significantly higher than in the control group (median 1.6 T cells/HPF) (Mann-Whitney U test, p = 0.01). T-cell infiltration at the periphery (median +/++ in both groups) did not show significant differences between the RIC and control groups (Mann-Whitney U test, p = 0.67).

**Figure 5:**
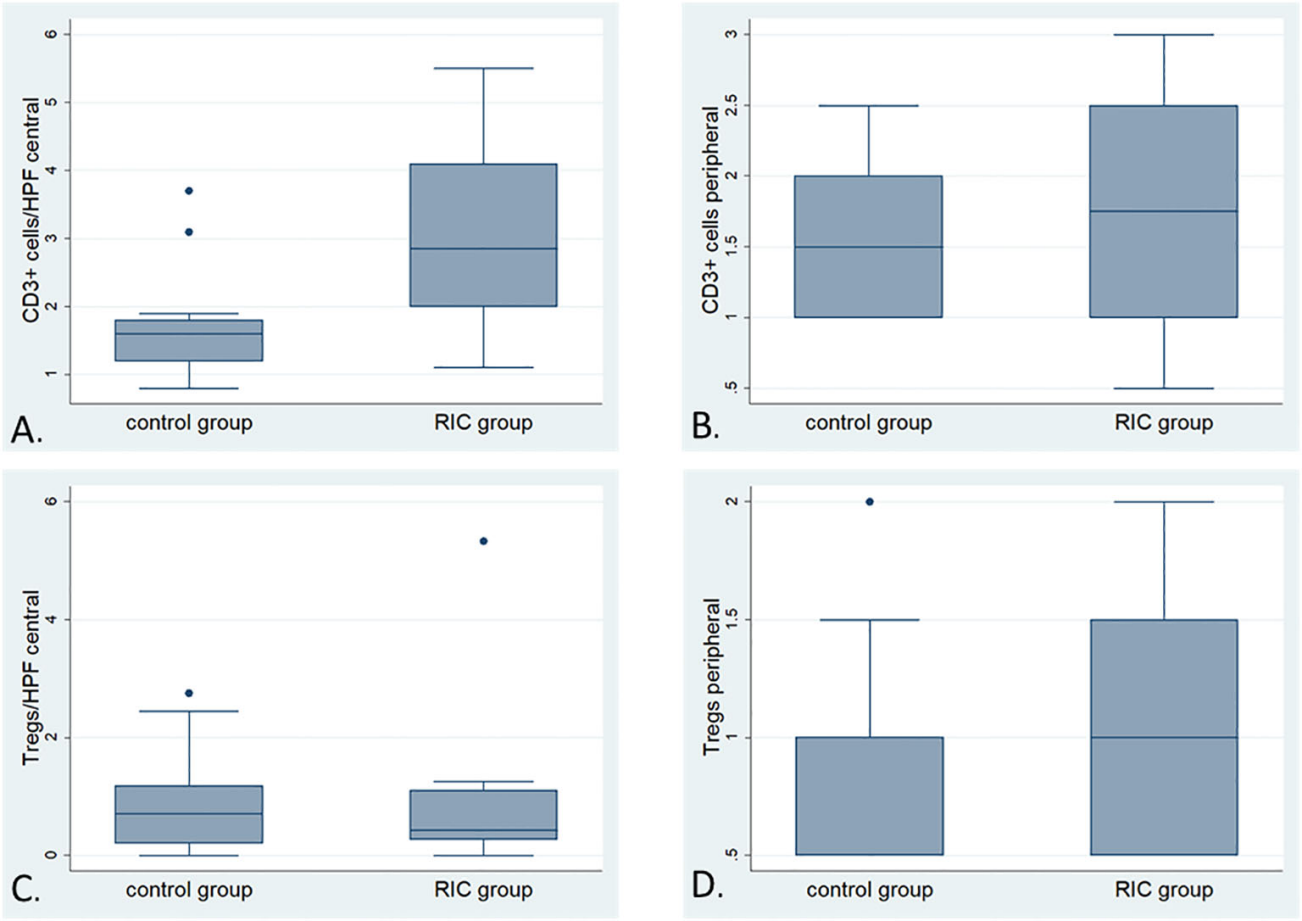
Box plots illustrating the detailed comparison of immune infiltration with CD-positive and FoxP3-positive cells (Tregs) in the RIC and control groups; **(A)** Comparison of the number of CD3-positive cells (average of cells counted from 10 randomly selected high-power fields at 400x magnification) in the tumor center between the RIC (median 2.8 T cells/HPF) and control groups (median 1.6 T cells/HPF). The immune infiltration with T cells in the tumor center appears to be more pronounced in the tumor center of the RIC group than in the control Mann-Whitney U test, p=0.01). **(B)** The box plots illustrate the comparison of immune infiltration with CD3-positive cells (analyzed using a semi-quantitative score) at the tumor periphery between the RIC (median “+/++”) and control groups (median “+/++”) (Mann-Whitney U test, p = 0.67). **(C)** Comparison of the number of FoxP3-positive cells (average of cells counted from 10 randomly selected high-power fields at 200x magnification) in the tumor center between the RIC [median 0,41/HPF (200x)] and control groups [median 0.71/HPF (200x)]. The immune infiltration with FoxP3-positive cells in the tumor center showed no significant differences between RIC and control groups (Mann-Whitney U test, p = 0.84). **(D)** The box plots illustrate the comparison of immune infiltration with Foxp3-positive cells (analyzed using a semi-quantitative score) at the tumor periphery between the RIC [(median 0.6/HPF (200x)] and control groups [(median 0.58/HPF (200x)], which is also not statistically significant (Mann-Whitney U test, p= 0.76).

In the RIC tumors, the median number of CD3- positive cells from 10 randomly selected high-power fields (HPF, 400 magnification) in the tumor center reached 2.8 T cells/HPF. It was significantly higher than the control group with 1.6 T cells/HPF (MWU, p=0.01) (**Figures 4**, **5A**). The median of peripheric infiltration with CD3-positive cells was +/++ in both groups, and the differences were insignificant (MWU, p= 0.67) (**Figures 4**, **5B**).

##### 3.2.3.2 FoxP3-positive cells (Tregs)

**Figure 6:**
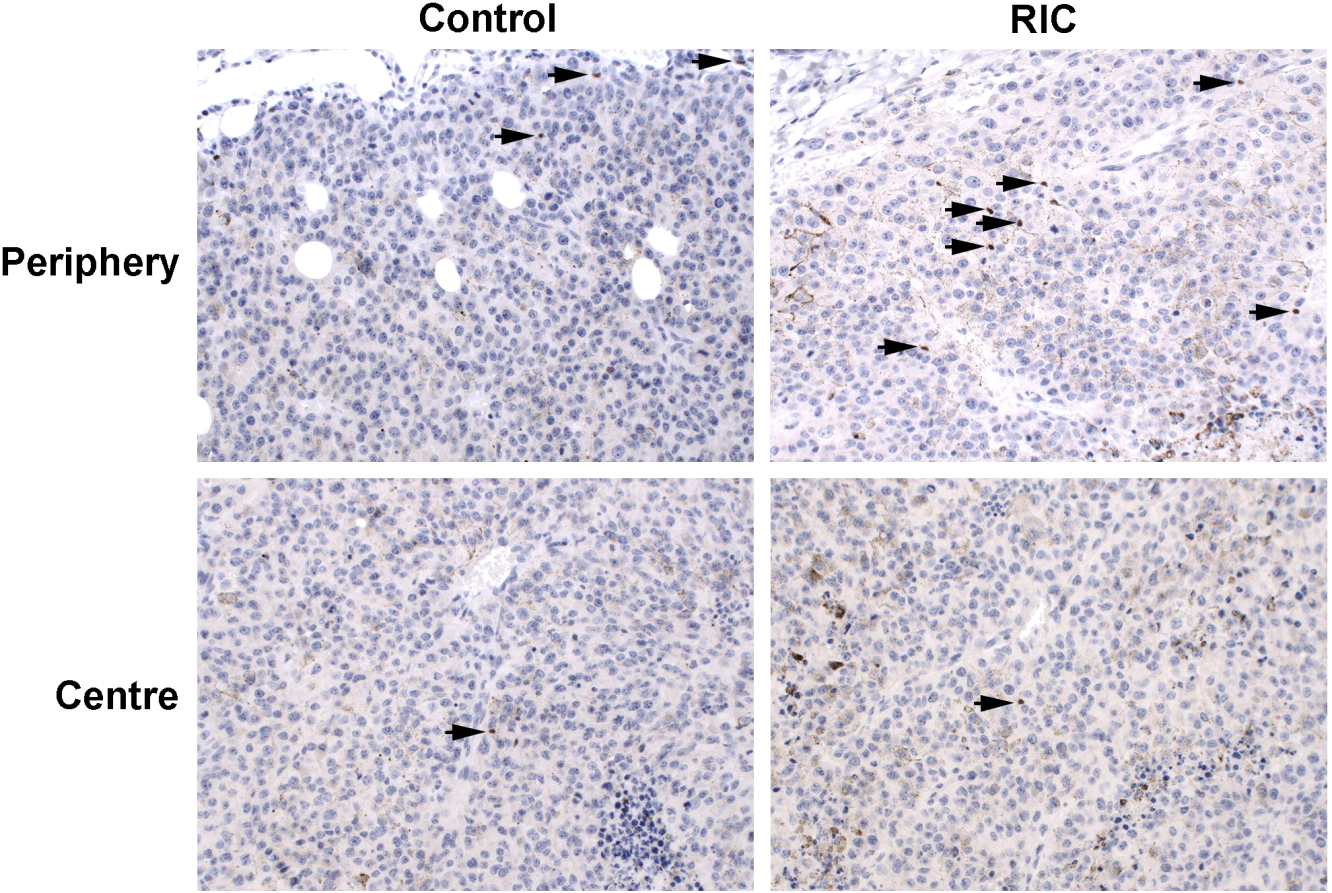
FoxP3 immunohistochemistry of tumor samples, presented at 200x magnification, identifies FoxP3-positive cells by brown staining and small cell nuclei, marked with black arrows. The immunohistopathological slides reveal low to mild (+/++) infiltration of Treg cells in the tumor periphery of the control group melanoma sample, with low (+) infiltration in the tumor center. Similarly, mild (++) infiltration of Treg cells was observed in the tumor periphery of the RIC group specimen, accompanied by low (+) infiltration in the tumor center. In the tumor center, the median number of Treg cells was 0.71/HPF (200x) in the control group and 0.41/HPF (200x) in the RIC group, which did not exhibit significant differences (Mann-Whitney U tests, p = 0.84). In the tumor periphery, the semiquantitatively analyzed number of Treg cells reached a median of “+” (mild infiltration) in both the control and RIC groups and showed no significant differences (Mann-Whitney U test; p = 0.63).

Overall, no differences were observed in terms of FoxP3-positive cells and their proportion to CD3-positive cells within the tumor and at the tumor periphery between the control and RIC groups. In the tumor center, the median number of Treg cells was 0.71/HPF (200x) (minimum = 0; maximum = 2.75) in the control group and 0.41/HPF (200x) (minimum = 0; maximum = 5.33) in the RIC group (MWU, p = 0.84) (**Figures 5C**, **6**). In the tumor periphery, semi-quantitative analysis of Treg infiltration showed a median score of 1 (+) in both the control and RIC groups, with identical minimum (0.5; -/+) and maximum values (2; ++). The difference between the groups was not statistically significant (MWU, p = 0.627) (**Figures 5D**, **6**).

The ratio of Tregs to the total immune infiltration was assessed as the ratio of FoxP3 (200x magnification) to CD3-positive cells (400x magnification) in the tumor center and periphery. In the RIC group, the median of this ratio reached 0.21 (minimum = 0; maximum = 1.13), and in the control group, it was 0.51 (minimum = 0; maximum = 1.45) in the tumor center (p = 0.19). In the tumor periphery, the median number of semiquantitatively analyzed Tregs reached 0.58 (minimum = 0.33; maximum = 1.5) in the control group and 0.6 (minimum = 0.2; maximum = 1.33) in the RIC group (p = 0.76). These differences were statistically non-significant.

### 3.3 Multiplex immunoassay

**Figure 7:**
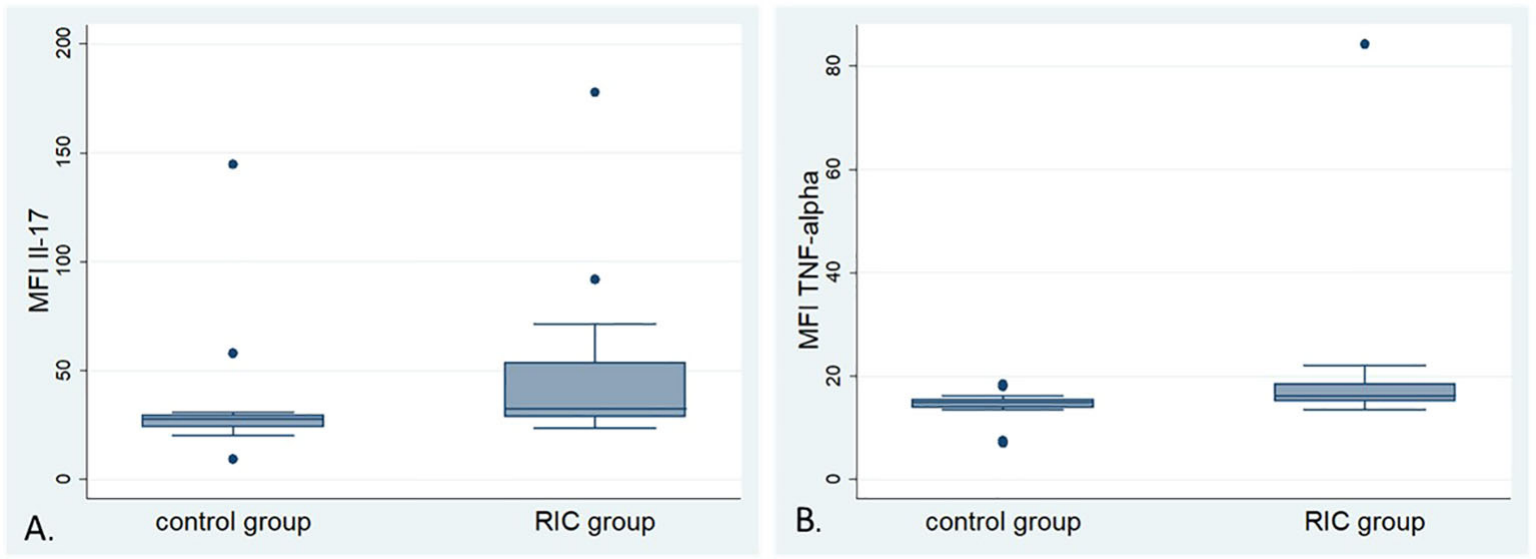
Box plots of the mean fluorescence intensity (MFI) of selected cytokines: **(A)** Box plots of the MFI of Interleukin-17 (IL-17) in the control and RIC groups. The MFI of IL-17 shows higher values in the RIC group compared to the control group (Mann-Whitney U-test, p = 0.035). **(B)** Box plots of the MFI of Tumor Necrosis Factor α (TNF-α) in the control and RIC groups. The MFI of TNF-α shows a tendency towards higher values in the RIC group than in the control group (Mann-Whitney U-test, p = 0.063).

23-cytokines: IL-1α, IL-1β, IL-2, IL-3, IL-4, IL-5, IL-6, IL-9, IL-10, IL-12 (p40), IL-12 (p70), IL-13, IL-17A, Eotaxin, G-CSF, GM-CSF, IFN-γ, KC, MCP-1, MIP-1α, MIP-1β, RANTES, and TNF-α were analyzed in 12 serum samples from the RIC and 13 samples from the control group. Three probes were excluded from the analysis due to hemolysis and/or inconsistent results in the analyzed duplicates. Relative median fluorescence intensities (MFI) from each assay were compared between the RIC and control groups (39). The MFI of the IL-17 in the RIC group was higher (median 34.4) than in the control group (median 28) (MWU, p=0.035) (**Figure 7A**). We observed a tendency toward a higher MFI of TNF- α in the RIC (median 16.3) when compared to the control group (median 15) (MWU, p=0.063) (**Figure 7B**). Other cytokines did not show relevant differences between both groups. There was no correlation between the MFIs of TNF-α and the number of CD3-positive cells in the tumor center (Pearson’s r = 0.13, p = 0.55) or the periphery (Spearman’s ρ = 0.08, p = 0.7). These correlations were also non-significant for IL-17 and T cells in the tumor center (Pearson’s r = 0.03, p = 0.9) and at the tumor periphery (Spearman’s ρ = -0.05, p = 0.8).

Furthermore, no significant correlation was observed between the MFI of IL-17 (Pearson’s r = -0.29, p = 0.15) or TNF-α (Pearson’s r = -0.11, p = 0.61) and Treg immune infiltration in the tumor center. Similarly, the semi-quantitative score of Treg infiltration at the tumor periphery did not correlate with the MFIs of IL-17 (Spearman’s ρ = -0.22, p = 0.30) or TNF-α (Spearman’s ρ = -0.23, p = 0.28).

## 4 Discussion

To our knowledge, no previous study has evaluated the effect of RIC on solid tumors. According to the metanalysis of Weir et al., the RIC effect provoked by the occlusion of the femoral artery in C57BL/6N mice models was neuroprotective in experimental ischemic stroke with the most effective total dose between 10 and 45 min and more than four cycles (42). Drysch et al. used the non-invasive low-pressure tourniquet with 0.6 N in the C57BL/6N strain (39). They showed that the low-pressure tourniquet causes a significant decrease in tissue blood flow and oxygenation, increased damage to muscle fibers, and increased apoptotic markers, which were superior to a clamping method, probably due to the closure of the collateral blood vessels with the tourniquet. Our rationale for conducting six RIC cycles with 30 minutes of ischemia using a non-invasive method is in line with the available literature.

Under isoflurane anesthesia in the control group, we observed a trend towards increased blood flow of the non-ischemic hind limb (p = 0.07), concurrent with improved oxygen saturation in the capillary-venous vascular bed (p = 0.06). Conversely, in the tumor tissue, only a significant increase in oxygen saturation in the capillary-venous vascular bed was noted (p = 0.035), with no changes in flow or hemoglobin content. These measurements could suggest an additional influence of isoflurane anesthesia on tumor and non-ischemic paw perfusion. Szczesny et al. described that anesthesia with evaporated isoflurane at concentrations of up to 4% during the induction phase, up to 1.5% during acute surgical procedures, and 0.8 - 1.3% during longer experimental observations was well-tolerated in murine models without respiratory complications or alterations in systemic circulatory parameters (43). The anesthesia protocol involving 4% isoflurane during induction and a maintenance dose of 0.8 - 1.0% was similarly applied in our experiment without complications. However, it has also been demonstrated that isoflurane had a cardiodepressive effect in C57BL/6N mice (44). In humans, Stevens et al. showed that isoflurane anesthesia could lead to vasodilation, reduced vascular resistance, lowered arterial blood pressure, and reduced cardiac output (45). However, they also observed a slight increase in blood flow in muscle and skin. The reduced cardiac output was initially compensated by an increase in heart rate during anesthesia. Higher concentrations of isoflurane (2.4%) resulted in reduced oxygen consumption. Nevertheless, Brett et al. observed other effects of isoflurane in newborn lambs, showing that isoflurane led to reduced regional blood flow in the brain, flank, heart, kidneys, adrenal glands, and spleen, accompanied by a more pronounced cardiodepressive effect characterized by reduced heart rate, cardiac output, and blood pressure (46). Systemic vascular resistance remained unchanged in newborn lambs, and oxygen extraction was only reduced at minimum alveolar concentration (MAC) of 1.5. Palmisano et al. examined the impact of isoflurane on isolated rabbit hearts and found a dose-dependent acceleration of atrioventricular conduction, an increase in coronary blood flow, and a dose-dependent reduction in oxygen extraction (47).

In our study, under isoflurane anesthesia in the control group, we observed a slight increase in blood flow (p = 0.07) and oxygen saturation (p = 0.06) in the hind paw, with an unchanged relative hemoglobin content. This could be explained by the mild vasodilatory effect of isoflurane, which led to a faster exchange of blood in the vascular bed of the hind paw, resulting in increased tissue oxygen saturation. Vessels in the tumor area responded differently to isoflurane anesthesia. Here, a statistically significant increase in oxygen saturation (p = 0.035) was observed without changes in flow or relative hemoglobin content. In the tumor tissue characterized by a pathological vascular system, isoflurane does not seem to have a significant vasodilatory effect. The sole increase in oxygen saturation of the tumor tissue may result from reduced oxygen extraction due to decreased tumor tissue metabolism during anesthesia. This effect of isoflurane anesthesia might occur preferentially in tumor tissue due to the higher basal metabolic rate of cancer cells. No significant differences were found in the intra-individual comparison of perfusion parameters between the baseline and final phases in both the non-ischemic hind limb and tumor tissue in the RIC group (p > 0.1). It can be noted that RIC was able to at least partially counteract the effects of isoflurane on tumor and hind paw perfusion in this comparison.

However, the tumor oxygen saturation in the RIC group showed a tendency towards improved tumor tissue oxygenation in the final phase. It was 11.3 percentage points (and thus 18%) higher than in the sham group in direct comparison (MWU, p = 0.09). It is also noteworthy that in the RIC group, tumor oxygen saturation relative to paw oxygen saturation was statistically significantly higher (MWU, p = 0.026) than in the control group. This suggests that RIC may have a different effect on the vascular beds of healthy tissue and tumors and can improve the relative oxygenation of tumor tissue. Kolbenschlag et al., using the same measurement method, observed that in free flaps, the application of RIC significantly improved tissue oxygen saturation on postoperative days 1 and 12, and blood flow significantly improved on postoperative days 5 and 12 (31). The authors initially observed an increase in tissue oxygen saturation, followed by a statistically significant improvement in blood flow after a few days. In the context of our findings, these observations may suggest that an initial increase in tumor tissue oxygen saturation relative to the control group could be a first step in the positive modification of microperfusion following short-term RIC application.

Although some short-term changes in microcirculation have been documented, no statistically relevant changes in acute hypoxia in relation to chronic, endogenous hypoxia were seen in the immunohistochemistry. However, Nordsmark et al. observed that the invasive tissue oxygenation measurements (invasive O_2_ sensitive electrodes) in human uterine cervix carcinomas did not clearly correlate with pimonidazole labeling due to a heterogenous distribution of hypoxic areas within the three-dimensional tumor probes (41).

The main changes induced by RIC in our study are related to the inflammatory response, as the number of CD3-positive tumor-infiltrating lymphocytes (TILs) in the tumor center (MWU, p = 0.01) was significantly higher than in the control group. Despite the increase in infiltration with TILs in the tumor center in the RIC group, the infiltration of Treg cells was comparable in both groups in the tumor center (MWU, p = 0.84) and the tumor periphery (MWU, p = 0.76). There was no correlation between the MFIs of cytokines and the number of TILs within or around the tumor. Considering that the increase in CD3-positive cells in the tumor center in the RIC group occurred in a brief timeframe, it is conceivable that this infiltration was influenced by the entry of already activated anti-tumoral T cells as part of the adaptive immune response. Generally, infiltration with T cells has been associated with better overall survival in human melanoma and a better response to immune therapies (16–18, 20, 22, 48). Vasaturo et al. observed that patients with metastatic melanoma who obtained dendritic cell vaccination had better overall survival (OS) when the infiltration of CD3-positive TILs was stronger (21). Moreover, they identified that the distribution of CD3-positive cells within the tumor (assessed by intratumoral versus peritumoral T-cell densities) was the most substantial predictive factor for OS after the treatment (21). In a comprehensive study on colorectal carcinoma by Galon et al., a positive correlation was observed between the presence of markers for Th1 polarization, cytotoxic T cells, and memory T cells, and a lower incidence of tumor recurrences (49). The type and density of immune cells in colon carcinomas have demonstrated prognostic value that surpasses the Union for International Cancer Control (UICC) TNM classification and is independent of it (49, 50).

The meta-analysis by Shang et al. confirmed that infiltration with FoxP3-positive-Tregs cumulatively correlated with a significantly lower survival rate across all cancer types [Odds Ratio (OR) of 1.46] (51). Another meta-analysis by Shou et al. demonstrated that a higher number of infiltrating FoxP3-positive T cells in breast cancer patients was associated with a lower overall survival rate and was significantly associated with a positive human epidermal growth factor receptor 2 (HER2) status, a positive lymph node status, and negative estrogen receptor (ER) status (52). FoxP3-blocking antisense oligonucleotides (ASOs) had already been tested in preclinical models and were able to decrease the number of infiltrating FoxP3-positive Tregs by 70% *in vitro* and *in vivo*, strongly modulated Treg effector molecules [e.g., Inducible T-cell Co-Stimulator (ICOS), CTLA-4, CD25, and tumor necrosis factor receptor superfamily member 9 (TNFRSF9)] increased the activation of CD8-positive T cells, and generated antitumoral activity in syngeneic tumor models (53). In clinical models, patients with advanced melanoma were shown to have a significantly higher proportion of circulating Tregs compared to those with minimal residual disease. The lack of clinical and immunological efficacy of the NY-ESO-1 ISCOMATRIX cancer vaccine was associated with an increased number of Treg cells in patients with advanced melanomas (54). Based on the existing evidence, it is anticipated that Tregs primarily exert a detrimental effect on the antitumor immune response. RIC appears to selectively induce an increase in immune infiltration with CD3-positive cells but not Tregs in the tumor center and may potentially enhance the antitumor response.

In the cytokine analysis, it was observed that the two groups differed in terms of the mean fluorescence intensity (MFI) of IL-17, with a higher MFI in the RIC group compared to the control group (MWU, p = 0.035) and a trend towards higher MFI of TNF-α in the RIC group (MWU, p = 0.063). In their murine melanoma model, Garcia-Hernandez et al. observed that the transfer of OVA-specific IL-17–producing CD8-positive T cells to melanoma-bearing mice suppressed tumor growth and led to enhanced recruitment of TILs, probably owing to the secretion of IL-17, TNF- α, and IFN-g by the transferred Tc17 (55). The described co-stimulatory effect of IL-17 and TNF α - resembles the cytokine signature in the RIC group in our study. On the other hand, neutralization of IL-17 or G-CSF and the absence of γδ T cells prevented neutrophil accumulation in the murine model of breast cancer, reduced metastasis rate, and inhibited the tumor’s progression (56). In another *in vivo* study, λ-carrageenan was injected directly into melanomas and inhibited local progression in mice bearing B16-F10 tumors (57). It also enhanced the immune response to tumors by increasing the presence of M1 macrophages, dendritic cells (DCs) and activated CD4(+)CD8(+) T lymphocytes within the spleen, augmented the secretion of IL17A in the spleen and significantly elevated TNF-α levels within the tumor. The injection of λ-carrageenan caused a significant increase in the production of anti-OVA antibodies (57).

Spontaneously mutated neoantigens in B16 melanomas are mostly insufficient to induce robust intratumoral immune cell infiltrates, and the initial addition of strong target model neoantigens (such as OVA) to B16 can enhance T-cell infiltration (58). Zelba et al. observed that the influence of IL-17 and T-cell responses in human melanoma highly depends on a targeted antigen (59). Patients with mainly circulating CD4-positive T cells against Melan-A had a worse OS than those with CD8-type response. Production of IL-17 by the CD4-positive T cells in these patients attributed independently to the worse prognosis. Patients with dominating T cells reactive to the NY-ESO-1 antigen had better-prolonged survival, independent of the type of T-cell response or IL-17 production.

TFN-α is a pleiotropic pro-inflammatory cytokine with diverse effects on cancer cells (60–70). TNF-α, through the activation of Tumor Necrosis Factor Receptor 1 (TNFR1) signaling, induces the upregulation of adhesion molecules on blood vessels and can prime and recruit CD8-positive T cells to infiltrate the tumor tissue via activation of Tumor Necrosis Factor Receptor 2 (TNFR2) (61, 69). Based on a preclinical murine model of colorectal tumors treated with CD4-positive T cell-based adaptive immunotherapy, Habtetsion et al. demonstrated that T cell-derived TNF-α can enhance oxidative stress and induce tumor cell death in a Nicotinamide Adenine Dinucleotide Phosphate Hydrogen (NADPH) oxidase-dependent manner (65). The reduction of oxidative stress through the inhibition of TNF-α signaling in tumor cells or through the scavenging of reactive oxygen species (ROS) antagonized the therapeutic effects of immunotherapy. On the other hand, TNF administration in the murine melanoma metastatic model increased metastasis and correlated with the infiltration of regulatory CD4(+)/Foxp3(+) T cells in the lungs (62).

In conclusion, both isoflurane anesthesia and RIC have an impact on microperfusion. Under isoflurane anesthesia, higher blood flow and tissue oxygenation were observed within the normal tissue (non-ischemic hind limb), which could be explained by a mild vasodilatory effect of isoflurane. Tumor tissue responded to isoflurane anesthesia with a significant increase in oxygen saturation alone, possibly resulting from reduced oxygen extraction due to decreased tumor tissue metabolism during anesthesia. The application of RIC counteracted some of these effects, primarily in healthy tissue, and led to a significant improvement in relative tumor tissue oxygenation compared to the non-ischemic hind limb. However, at the end of the experiment, there was a slight tendency toward better tumor oxygen saturation in a direct comparison between the control and RIC groups.

RIC primarily exhibited a positive immunomodulatory effect on tumors and selectively enhanced immune infiltration within the tumor, particularly by previously activated CD3-positive T cells, while having no significant impact on T-regulatory cells. RIC seems to influence the cytokine profile (with higher MFIs of TNF-α and IL-17). Still, a balance between high and low concentrations of IL-17 and TNF-α can exert opposing effects on tumor growth in various models, warranting further investigation. Additionally, the repetitive application of RIC during radio-, chemotherapy or immunotherapy with various numbers of RIC, as well as the long-term effect of RIC on the antitumoral response, should be further investigated.

A limitation of this study is that the B16 tumor model does not capture the genetic diversity of human melanoma cells and skips the step of tumorigenesis (71). B16-OVA cells carry a non-physiological antigen, ovalbumin, which can stimulate the host immune system’s recognition more robustly than antigens found in human melanoma. This study faces limitations due to the intrinsic complexities associated with employing an unbiased murine model. Such models, while valuable, encounter significant challenges in mirroring the multifaceted nature of real-world conditions, where a multitude of interdependent variables create unpredictable dynamics. Consequently, the study’s findings may lack the nuanced fidelity necessary to fully capture the elaborate immune responses characteristic of human melanoma, underscoring the translational gap between controlled animal models and the complexity of human immunopathology.

Due to the diffuse nature of hypoxia, the assessment of hypoxic areas could only be conducted using a semiquantitative score. While immunohistochemistry proved valuable in our study of the immune response in a murine melanoma model, several limitations should be acknowledged. The qualitative nature of immunohistochemistry analysis can lead to subjective interpretations of staining patterns. This variability in scoring by different observers may introduce bias, impacting the conclusions drawn regarding immune cell infiltration. Another important limitation is the semi-quantitative assessment characteristic of immunohistochemistry. While it provides insights into the presence and localization of immune markers, it often lacks precise quantitative capabilities. Moreover, immunohistochemistry offers only a static view of protein expression at a specific time point, meaning it cannot capture dynamic changes in immune cell activation or migration over time. This limitation restricts our understanding of the temporal aspects of the immune response in melanoma progression. Furthermore, the strong pigmentation of the tumors might impact the O2C measurements.

## Data Availability

The raw data supporting the conclusions of this article will be made available by the authors, without undue reservation.
